# Effect of acacia polyphenol on glucose homeostasis in subjects with impaired glucose tolerance: A randomized multicenter feeding trial

**DOI:** 10.3892/etm.2013.1029

**Published:** 2013-03-26

**Authors:** SOSUKE OGAWA, TOMOYUKI MATSUMAE, TAKESHI KATAOKA, YOSHIKAZU YAZAKI, HIDEYO YAMAGUCHI

**Affiliations:** 1 mimozax Co., Ltd., Hatsukaichi-shi, Hiroshima 738-0034, Japan;; 2 Department of Chemical Engineering, Monash University, Clayton, Victoria 3800, Australia;; 3 Research Center, Total Technological Consultant Co., Ltd., Shibuya-ku, Tokyo 150-0021, Japan

**Keywords:** dietary supplement, acacia polyphenol, impaired glucose tolerance, glucose homeostasis, insulin, oral glucose tolerance test

## Abstract

Numerous *in vitro* and animal studies, as well as clinical trials have indicated that plant-derived polyphenols exert beneficial effects on glucose intolerance or type 2 diabetes. This clinical study aimed to investigate the effects of acacia polyphenol (AP) on glucose and insulin responses to an oral glucose tolerance test (OGTT) in non-diabetic subjects with impaired glucose tolerance (IGT). A randomized, double-blind, placebo-controlled trial was conducted in a total of 34 enrolled subjects. The subjects were randomly assigned to the AP-containing dietary supplement (AP supplement; in a daily dose of 250 mg as AP; n=17) or placebo (n=17) and the intervention was continued for 8 weeks. Prior to the start of the intervention (baseline) and after 4 and 8 weeks of intervention, plasma glucose and insulin were measured during a two-hour OGTT. Compared with the baseline, plasma glucose and insulin levels at 90 and/or 120 min, as well as the total area under the curve values during the OGTT (AUC_0→2h_) for glucose and insulin, were significantly reduced in the AP group, but not in the placebo group after intervention for 8 weeks. The decline from baseline in plasma glucose and insulin at 90 or 120 min of the OGTT for the AP group was significantly greater compared with that of the placebo group after 8 weeks of intervention. No AP supplement-related adverse side-effects nor any abnormal changes in routine laboratory tests and anthropometric parameters were observed throughout the study period. The AP supplement may have the potential to improve glucose homeostasis in subjects with IGT.

## Introduction

The prevalence of type 2 diabetes is rising exponentially and has become a global health priority (1,2). The International Diabetes Federation has predicted that the number of individuals with diabetes is likely to increase from 240 million in 2007 to 380 million in 2025 (1,3). More than 60% of the world’s population with diabetes are likely to come from Asia since this geographic region remains the world’s most populated area (4). It is also noted that the number of individuals with diabetes and impaired glucose tolerance (IGT) in each Asian country, including Japan, is likely to increase sustainedly in coming decades.

Currently, the prevention of type 2 diabetes in subjects with IGT involves lifestyle modifications, including increased physical activity and weight control by reduced caloric intake (5,6). Moreover, the dietary recommendations for individuals who are at risk of type 2 diabetes emphasize the intake of various plant food products, including whole grains, berries, fruits and vegetables, all known to be not only excellent sources of dietary fiber, but also good sources of variable polyphenolic compounds as reviewed by Hanhineva *et al*(7). These compounds affect glucose metabolism by several different mechanisms, including inhibition of carbohydrate digestion and glucose absorption in the intestine, stimulation of glucose release from the liver, activation of insulin receptors and glucose uptake in the insulin-sensitive tissues and modulation of hepatic glucose output.

An essential group of polyphenolic compounds are flavonoids encompassing various structural classes, including flavones, flavonols, flavans, flavanones, anthocyanins, chalcones, aurones and isoflavones. Representing the most common flavonoids consumed in the diet, flavan-3-ols have been reported to exhibit several health-beneficial effects by acting as antioxidant, anticarcinogenic, cardiopreventive, antimicrobial, antiviral and neuroprotective agents (8).

*Acacia mearnsii* (black wattle) grows naturally on the mainland of south-eastern Australia and in Tasmania. *A. mearnsii* was first introduced from Australia to South Africa in 1864 and since then its plantations have been established not only in South Africa, but also in eastern Africa and South America (9). The aqueous extracts of the bark of *A. mearnsii* contain significant amounts of polyphenols, referred to as acacia polyphenol (AP), whose major components are unique flavan-3-ol oligomers and polymers consisting of 4 or 5 monomeric units, including robinetinidol, fisetinidol, catechin and gallocatechin (10–14). In Japan, a tablet-form product of AP preparation, which contains ∼80% (w/v) polyphenols with molecular weights ranging from 300 to 3,000 kDa (15,16), is commercially available. With this AP-containing supplementary diet product (referred to as AP supplement), Ikarashi *et al* conducted animal studies using KKAy mice, model animals for obesity and type 2 diabetes, to evaluate its anti-obesity and antidiabetic effects, and identified that oral doses of the AP supplement significantly inhibited body weight gain and reduced hyperglycemia and insulin resistance (17,18). Our previous clinical study demonstrated that healthy adults had a high tolerance for the 4-week intake of AP supplement in daily doses ≤1,000 mg AP (19).

The promising results of these animal and human studies led us to conduct a clinical trial to evaluate the effect of short-term intake of this AP supplement on glucose and insulin responses to an oral glucose tolerance test (OGTT) in non-diabetic adult subjects presenting IGT.

## Subjects and methods

### Subjects

Male and female Japanese participants with diagnosed IGT, aged 20–70 years, were included in this study. The diagnosis of IGT was made by an OGTT with a 250–300 ml solution of 75 g anhydrous glucose. Plasma glucose at 120 min after loading was ≥140 and <200 mg/dl according to the Guidelines for the Treatment of Diabetes 2001–2003 (20) and the American Diabetes Association criteria for IGT (21). Participants were excluded if they had clinically overt diabetes with current 2-h plasma glucose levels in a 75 g OGTT >200 mg/dl and hemoglobin Alc (HbAlc) levels >6.5% or if they were receiving antidiabetic, antihyperlipidemic and/or antihypertensive treatment(s) or other medications that may have affected their glucose metabolism, lipid metabolism and/or blood pressure. Participants were also excluded if they currently suffered from familial hyperlipidemia, chronic renal failure, cardiovascular dysfunction or systemic infection or had a past history of such medical conditions and/or any other serious diseases. The other exclusion criteria were: routine intake of antioxidant health food or red wine; participation in another clinical study at the start time of the present study; known allergies to any food or medicine; being a pregnant woman, nursing mother or a woman of childbearing potential; and the presence of any clinically significant medical condition judged by the investigator to preclude the participant’s inclusion in the study.

### Study design

A randomized, multicenter, placebo-controlled study was designed to assess the efficacy and safety of the AP supplement for improving the IGT in enrolled subjects when compared with the placebo. The study was performed from June 2006 to October 2006 and involved five clinical service organization centers in Japan. The study protocol was approved by the institutional ethics committees. The study was conducted in accordance with the principles of the Declaration of Helsinki in 1995 (as revised in Edinburgh, 2000) and the Ethical Guidelines for Epidemiological Research (enacted by the Japanese Government in 2004). Written informed consent was obtained from all participants prior to their enrollment in the study. The overall design of the study consisted of an 8-week intervention period preceded by a ∼4-week run-in period, during which eligible subjects were screened.

### Intervention and subject assignment

The AP supplement used in this study was a 300 mg tablet preparation containing 250 mg AP in a daily dose of four tablets. The AP sample was prepared from hot-water extracts of the bark of *A. mearnsii* that was derived from a plantation in South Africa. As shown in Table I, there was no nutritionally significant difference in carbohydrate content (and thus in energy) between the AP tablet and the AP-free placebo tablet. In addition, the AP and placebo tablets were similar in color and packaging.

The study intervention started ∼1 week after screening of eligible subjects. The subjects were sequentially assigned based on random number tables to one of the two masked products and randomized (1:1) to the AP supplement (AP group) and the placebo (placebo group). All subjects were required to take four tablets of the allocated product over the 8-week intervention period, to accomplish full clinical and laboratory examinations at the start of intervention (baseline) and after 4 and 8 weeks intervention and to self-record their intake of the allocated tablet and occurring adverse events in a study diary. In addition, subjects were instructed to maintain their usual diet and to continue their usual physical activity behaviors throughout the intervention.

### OGTT results-based efficacy assessment

To evaluate the effectiveness of the AP supplement in improving IGT, an OGTT was performed in each subject at the start of intervention (baseline) and after 4 and 8 weeks of intervention, following a 10–14 h overnight fast and ≥12 h from the last study tablet intake. Plasma glucose and insulin concentrations at 0, 30, 60, 90 and 120 min after the glucose load were determined to assess efficacy upon glucose metabolism. The 2-h area under the curve (AUC_0→2h_) values for glucose and insulin were the additional measures for efficacy assessment. The OGTT values for plasma levels of glucose and insulin at 0 min (G_0_ and I_0_, respectively) were also utilized for measurement of an index of insulin resistance, the homeostasis model assessment of insulin resistance (HOMA-IR), calculated as follows: HOMA-IR = G_0_ (mg/dl) × I_0_(*μ*U/ml)/405 (20,22–25).

### Measurement of hematochemical, hematological and anthropometric parameters

Total cholesterol, low-density lipoprotein (LDL)-cholesterol, high-density lipoprotein (HDL)-cholesterol, triglycerides, serum electrolytes and other routine hematochemical laboratory test variables, including total protein, albumin, alkaline phosphatase, lactate dehydrogenase, aspartate aminotransferase, alanine aminotransferase, γ-glutamyltranspeptidase, urea nitrogen, uric acid and creatinine, were measured in serum samples collected from individual subjects after an overnight fast at baseline and at 4 and 8 weeks after the start of treatment. HbA1c, red blood cell count, white blood cell count, platelet count, hemoglobin and hematocrit were measured in whole blood. In addition, several anthropometric parameters, including body mass index (BMI), blood pressure values and heart rate were also measured at the same time points during the study period.

### Safety assessment

Safety was assessed on the basis of the incidence and severity of intervention-related adverse events reported throughout the 8-week intervention period, as well as abnormal changes in hematochemical and hematological laboratory test variables and those in anthropometric parameters.

### Statistical analysis

The data were analyzed with PASW Statistics 18 (SPSS, Inc., Chicago, IL, USA). Continuous normally distributed data are expressed as means ± standard error of the mean (SEM). The significance of differences in mean values and proportions of parameters between the test (AP) and placebo groups was assessed by the Student’s unpaired t-test and the Chi-square test, respectively. Changes from baseline were calculated by subtracting the value at the baseline from the value after the 4- or 8-week intervention. The Student’s paired t-test was used to assess within-group differences between the values at the baseline and those after the 4- or 8-week intervention. Plasma glucose and insulin concentrations were measured at all time points (0, 30, 60, 90 and 120 min) during the OGTT. AUC_0→2h_ values for glucose and insulin were calculated using the trapezoid rule. P<0.05 was considered to indicate a statistically significant difference.

## Results

### Baseline characteristics of the study population

A total of 34 subjects were eligible. Seventeen subjects were assigned to each of the AP supplement (AP group) and the placebo (placebo group), and all of the subjects completed the entire study. The baseline characteristics of the two study groups are presented in Table II. The data shown in Table II were obtained on the first study day after the run-in period was completed. All subjects met the IGT inclusion criteria and none had diagnosed diabetes or insulin resistance when assessed on the basis of the fast plasma glucose concentration, serum HbA1c, 2-h plasma glucose level in 75 g OGTT and HOMA-IR determined at the end of the 2-week run-in. The HbA1c values and systolic and diastolic blood pressures were within the normal range in all the enrolled subjects, although a number of patients had a marginally high BMI and/or serum triglycerides.

At baseline, although mean values of almost all clinical variables, with the exception of the 2-h plasma glucose level in the OGTT in the two groups, were within the normal range, the mean values of serum triglycerides for the AP group (191.8±24.3 mg/dl) were above the normal range. Significant differences between the two groups were observed in mean values of age and serum triglycerides (P<0.05; Table II).

### Effect on IGT

**Figure 1:**
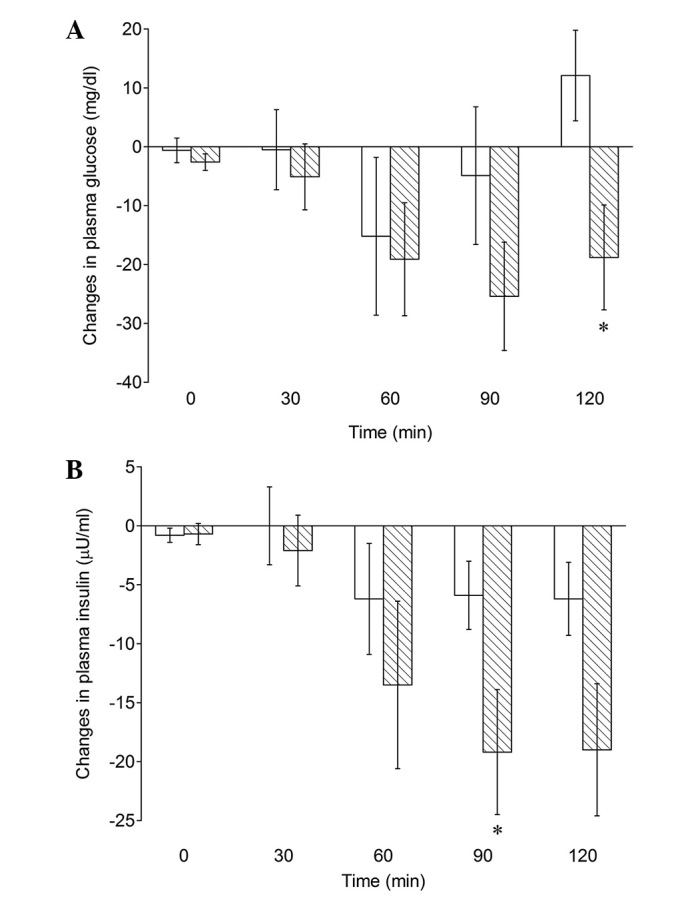
Comparison of changes from the baseline in plasma concentrations of (A) glucose and (B) insulin at 0, 30, 60, 90 and 120 min during a 2-h OGTT between the placebo group (open bars; n=17) and the AP group (diagonally striped bars; n=17) after intervention for 8 weeks. Data are presented as the mean ± standard error of the mean (SEM). ^*^P<0.05 vs. the placebo value. OGTT, oral glucose tolerance test; AP, acacia polyphenol.

Table III shows the plasma glucose response to a 2-h OGTT at baseline and after 4- and 8-week interventions. In the placebo group, there was no statistically significant difference in the mean value of plasma glucose concentrations at each time point of the OGTT from 0 to 120 min throughout the 8-week intervention period. By contrast, the mean values at 90 and 120 min for the AP group were significantly or near-significantly reduced after the 8-week intervention, compared with the baseline value (P=0.014 and 0.051, respectively). Moreover, as shown in Fig. 1A, there appeared to be clear differences in changes of plasma glucose from baseline at 90 and 120 min of the OGTT after the 8-week intervention between the AP and placebo groups, with the 120 min values reaching a statistical significance (−18.8±8.9 mg/dl vs. 12.1±7.7 mg/dl; P=0.013).

Intake of the AP supplement for 8 weeks also induced a similar downward trend in plasma insulin at 90 and 120 min of the OGTT (Table IV); mean plasma insulin concentrations at 90 and 120 min were significantly lowered compared with the baseline figures (P=0.002 and 0.004, respectively). Fig. 1B shows that there were significant differences in changes of plasma insulin from baseline for the 90 min value between the AP and placebo groups (−19.2±5.3 vs. −5.9±2.9 *μ*U/ml; P=0.032).

Moreover, the 8-week intake of the AP supplement significantly lowered the glucose and insulin AUC_0→2h_ values compared with the baseline levels (P=0.018 and 0.009, respectively), although there was no significant difference in changes of AUC_0→2h_ values for glucose or insulin from base-line between the AP and placebo groups (Table V). These results indicate that plasma glucose and insulin responses to the OGTT may be improved by the intake of an AP supplement for 8 weeks.

### Effect on other glucose metabolism-related variables

Fasting plasma glucose and insulin concentrations shown as the values at 0 min of the OGTT before the start of intervention were not significantly affected by the 4- or 8-week intake of the AP supplement (Tables III and IV). Similarly, the insulin resistance index, HOMA-IR, as well as HbA1c concentrations, were also scarcely altered during intervention in the AP and placebo groups (Table VI).

### Safety assessment

Two subjects in the placebo group and no subjects in the AP group reported adverse events. The correlation of adverse events with the allocated tablet was not ruled out during the study period. One of the two adverse events reported from the placebo group was gastralgia and the other was allergic symptoms. The two events were mild in severity and occurred only temporarily. In the two groups, routine laboratory tests and anthropometric parameters (body weight, BMI, blood pressure and pulse rate) did not show any significant abnormalities throughout the duration of the intervention.

## Discussion

IGT, as well as insulin resistance, is known to be associated with an increased risk of type 2 diabetes and hypertension, which are well-recognized risk factors for cardiovascular diseases (26–28). Considering the heavy burden of these metabolic disorders on the public health, improvement of IGT and/or insulin resistance is a supremely important health issue. It has been reported from animal experiments, in which KKAy mice with high-fat diet-induced obesity were used, that AP reduces hyperglycemia and hyperinsulinemia by increasing adiponectin secretion and suppressing TNF-α secretion by white adipocytes, as well as by enhancing the expression of GLUT4 in skeletal muscle. AP was also effective in decreasing the HOMA-IR value to a significant level (17). In consistent with this animal study, the present randomized, placebo-controlled trial in otherwise healthy subjects with IGT demonstrated that AP supplement intake for up to 8 weeks significantly reduced (improved) overall glucose and insulin responses to an oral glucose load in the OGTT, indicating a beneficial effect of the AP supplement on glucose homeostasis. When comparing the AP supplement with the placebo, there was no difference in the early plasma glucose and insulin responses, as presented by the similar mean incremental glucose values for the two study groups at 30 min after the start of OGTT. The major effects of the AP supplement were observed at 90 or 120 min, where there were significant reductions in plasma glucose and insulin relative to the placebo. By contrast, the AP supplement did not show any recognizable effects on plasma glucose nor insulin within the first 30 min. Thus, it is likely that, in our study designed to investigate the chronic effect of the AP supplement, its intake may be implicated in the improvement of glucose metabolism rather than the inhibition of glucose absorption from the small intestine.

In contrast to the positive effect on glucose intolerance, AP supplement intake did not alter the HOMA-IR value. This may be explained, partly at least, by the finding that the mean HOMA-IR values for the AP and placebo groups at baseline (1.2±0.1 and 1.6±0.2, respectively) were within the normal range (20), implying that the majority of subjects did not have insulin resistance (26,29–31).

Promising results concerning the effects of various dietary polyphenols, including flavonoids, phenolic acid, proanthocyanidins and resveratrol, on carbohydrate homeostasis have been obtained from numerous *in vitro* and animal studies as previously documented (7). However, there are extremely few controlled studies investigating the positive effects of specific polyphenols. Among them is the present study, which demonstrated that AP may have favorable metabolic effects and thereby further prevent the development of type 2 diabetes and, ultimately, cardiovascular diseases. Currently, the relevant mechanisms underlying the beneficial effects of AP on glucose metabolism are difficult to postulate since the molecular mechanism have not been comprehensively studied. For an improved understanding of the role of AP in the regulation of glucose metabolism, further studies assessing its effects on insulin and other hormonal responses are required.

Throughout the 8-week intervention period, no AP supplement-related adverse events were reported. The safety of the AP supplement is supported by our previous study demonstrating that a 4-week intake of the AP supplement in daily doses ≤1,000 mg AP was safe in healthy male adults (19). Further clinical trials are warranted to confirm the potential nutritional usefulness of the AP supplement in the improvement of glucose intolerance in populations affected by IGT or insulin-resistant conditions, including hyperglycemia and hyperinsulinemia, as well as obesity, hypertension and hyper-lipidemia.

Based on the results from the current study on efficacy and safety assessments, we conclude that the AP supplement may be safely administered and may improve glucose homeostasis in non-diabetic subjects with IGT.

## Figures and Tables

**Table I:** Composition of the AP and placebo tablets used in the present study.

Composition	Amount per daily dose (mg)	

	AP tablet	Placebo tablet
AP	250.1	0
Sugar esters^a^	36.0	36.0
Dextrin^a^	360.0	480.0
Reduced maltose^a^	180.0	180.0
Cellulose	353.3	442.2
Caramel^a^	20.6	61.8
Total carbohydrates	596.6	757.8

a Carbohydrates as the potential source of glucose and energy. AP, acacia polyphenol.

**Table II:** Baseline characteristics of 34 subjects in the two study groups who completed the entire study.

Clinical variable	Placebo group (n=17)	AP group (n=17)	P-value
Gender (male/female; n)	13/4	13/4	
Age (years)	55.1±2.0	49.6±1.5	0.038
Height (cm)	167±3	166±2	0.822
Weight (kg)	69.3±1.8	69.8±2.8	0.882
BMI (kg/m^2^)	24.8±0.6	25.1±0.8	0.765
Fasting plasma glucose (mg/dl)	114±2	112±3	0.631
Fasting plasma insulin (*μ*U/ml)	4.4±0.4	5.7±0.6	0.080
2-h plasma glucose level in 75 g OGTT (mg/dl)	181±9	173±9	0.545
HOMA-IR	1.2±0.1	1.6±0.2	0.083
Hemoglobin A1c (%)	5.5±0.1	5.5±0.1	0.635
Systolic blood pressure (mmHg)	122.4±4.4	125.2±2.3	0.565
Diastolic blood pressure (mmHg)	76.7±2.7	81.5±2.1	0.171
Total cholesterol (mg/dl)	201.9±8.5	208.2±8.2	0.593
LDL-cholesterol (mg/dl)	135.1±7.1	135.3±8.3	0.983
HDL-cholesterol (mg/dl)	50.5±3.3	49.8±3.0	0.886
Triglycerides (mg/dl)	135.3±12.7	191.8±24.3	0.048

All values are expressed as the mean ± standard error of the mean (SEM), with the exception of gender. The P-value was assessed by the Student’s unpaired t-test. AP, acacia polyphenol; BMI, body-mass index; OGTT, oral glucose tolerance test; HOMA-IR, homeostasis model assessment of insulin resistance; LDL, low-density lipoprotein; HDL, high-density lipoprotein.

**Table III:** Plasma glucose concentrations during an OGTT conducted immediately prior to the start of intervention (baseline) and after 4 and 8 weeks of intervention with the placebo (placebo group) or the AP supplement (AP group).

Study group	Intervention period	Plasma glucose (mg/dl)				

		0 min	30 min	60 min	90 min	120 min
Placebo (n=17)	Baseline	114±2	199±8	234±12	214±13	181±9
	4 weeks	117±3 (0.109)	205±9 (0.281)	229±12 (0.550)	220±14 (0.313)	190±12 (0.340)
	8 weeks	113±3 (0.761)	199±7 (0.945)	219±12 (0.272)	209±14 (0.682)	193±10 (0.137)
AP (n=17)	Baseline	112±3	185±7	221±10	206±10	173±9
	4 weeks	110±2 (0.204)	190±6 (0.269)	220±10 (0.965)	201±9 (0.666)	162±10 (0.202)
	8 weeks	110±2 (0.069)	180±6 (0.379)	202±11 (0.065)	181±12 (0.014)	154±9 (0.051)

All values are expressed as the mean ± standard error of the mean (SEM). The P-value of a within-group difference in measurement from baseline was assessed by the Student’s paired t-test and is shown in parentheses. Baseline values were determined prior to the start of intervention. OGTT, oral glucose tolerance test; AP, acacia polyphenol.

**Table IV:** Plasma insulin concentrations during an OGTT conducted prior to the start of intervention (baseline) and after 4 and 8 weeks of intervention with the placebo (placebo group) or the AP supplement (AP group).

Study group	Intervention period	Plasma insulin (*μ*U/ml)				

		0 min	30 min	60 min	90 min	120 min
Placebo (n=17)	Baseline	4.4±0.4	18.0±2.0	29.4±4.3	31.2±3.2	31.2±3.7
	4 weeks	5.5±0.8 (0.159)	23.2±3.1 (0.042)	35.9±7.0 (0.230)	37.9±5.7 (0.092)	40.2±6.8 (0.090)
	8 weeks	3.6±0.5(0.192)	18.1±3.8 (0.989)	23.2±4.5 (0.208)	25.5±4.4 (0.071)	25.0±3.3 (0.063)
AP (n=17)	Baseline	5.7±0.6	23.3±3.0	42.5±7.3	50.5±8.2	51.4±9.1
	4 weeks	6.5±0.9 (0.449)	29.0±4.4 (0.062)	47.2±8.9 (0.499)	60.7±10.6 (0.080)	52.4±7.7 (0.822)
	8 weeks	5.0±0.9 (0.472)	21.1±3.9 (0.489)	29.0±5.4 (0.075)	31.3±6.5 (0.002)	32.4±5.1 (0.004)

All values are expressed as the mean ± standard error of the mean (SEM). The P-value of within-group differences in measurement from baseline was assessed by the Student’s paired t-test and shown in parentheses. Baseline values were determined prior to the start of intervention. OGTT, oral glucose tolerance test; AP, acacia polyphenol.

**Table V:** Plasma glucose and insulin responses to an OGTT conducted prior to the start of intervention (baseline) and after 8 weeks of intervention with the placebo (placebo group) or the AP supplement (AP group) as revealed by glucose AUC_0→2h_ and insulin AUC_0→2h_.

Variable	Intervention period	Measures of variance		Between-group difference in variance (P-value)

		Placebo group (n=17)	AP group (n=17)	
Glucose AUC_0→2h_ (mg/dl/h)	Baseline	397.2±17.1	377.2±14.3	
	8 weeks	389.7±17.7 (0.622)	347.1±14.2 (0.018)	
	Change from baseline	−7.4±14.8	−30.1±11.4	0.234
Insulin AUC_0→2h_ (*μ*U/dl/h)	Baseline	48.2±5.0	72.4±11.0	
	8 weeks	40.5±6.8 (0.159)	50.0±8.5 (0.009)	
	Change from baseline	−7.6±5.2	−22.3±7.6	0.119

All values are expressed as the mean ± standard error of the mean (SEM). P-value of within-group differences in measurement from baseline was assessed by the Student’s paired t-test and shown in parentheses. Baseline values were determined prior to the start of intervention. OGTT, oral glucose tolerance test; AP, acacia polyphenol; AUC, area under the curve.

**Table VI:** HOMA-IR and hemoglobin Alc values determined prior to the start of intervention (baseline) and after 4 and 8 weeks of intervention with the placebo (placebo group) or the AP supplement (AP group).

Parameter	Intervention period	Measures of variance		Between-group difference in variance (P-value)

		Placebo group (n=17)	AP group (n=17)	
HOMA-IR	Baseline	1.2±0.1	1.6±0.2	
	4 weeks	1.6±0.3 (0.144)	1.7±0.2 (0.606)	
	Change from baseline	0.4±0.2	0.1±0.3	0.503
	8 weeks	1.0±0.1 (0.606)	1.4±0.2 (0.349)	
	Change from baseline	−0.2±0.2	−0.3±0.3	0.924
Hemoglobin Alc (%)	Baseline	5.5±0.1	5.6±0.1	
	4 weeks	5.5±0.1 (0.119)	5.4±0.1 (0.396)	
	Change from baseline	0	−0.1±0.1	0.931
	8 weeks	5.6±0.1 (0.154)	5.4±0.1 (0.318)	
	Change from baseline	0.1±0.0	−0.1±0.1	0.111

All values are expressed as the mean ± standard error of the mean (SEM). The P-value of within-group differences in measurement from baseline was assessed by the Student’s paired t-test and shown in parentheses. HOMA-IR, homeostasis model assessment of insulin resistance; AP, acacia polyphenol.
